# Dual computational systems in the development and evolution of mammalian brains

**DOI:** 10.1126/sciadv.aec6112

**Published:** 2026-04-22

**Authors:** Nabil Imam, Matthew Kielo, Brandon M. Trude, Barbara L. Finlay

**Affiliations:** ^1^ School of Computational Science and Engineering, Georgia Institute of Technology, Atlanta, GA, USA.; ^2^ Behavioral and Evolutionary Neuroscience Group, Department of Psychology, Cornell University, Ithaca, NY, USA.

## Abstract

Analyses of brain sizes across mammalian taxonomic groups reveal a consistent pattern of covariation between major brain components, including a robust inverse relationship between the limbic system and the neocortex. To find the functional basis of this relationship, we mapped the multidimensional representations of task-optimized artificial neural networks onto two-dimensional surfaces resembling the forebrain cortices. We found that networks optimized for visual, somatosensory, and auditory representations develop ordered spatiotopic maps where units draw information from localized regions of the sensory input. In contrast, networks optimized for olfactory and relational memory representations develop fractured maps with distributed patterns of information convergence. Evolutionary optimization of multimodal networks for varying task objectives results in inverse covariation between spatiotopic and disordered network components that compete for the representational space. These results suggest that the observed pattern of covariation between brain components reflects an essential computational duality in brain evolution.

## Teaser

Localized and distributed inductive biases shape the macroscale evolution of brain networks.

## INTRODUCTION

One neuroanatomical division of the vertebrate brain has attracted the attention of researchers for decades but has resisted functional definition or even a specific list of its contents. The “limbic system,” from “limbus” or “edge,” refers to a collection of forebrain parts derived from a common embryonic source. Its components are networks of varying complexity and size, principally the amygdala, septum, and other basal forebrain nuclei, and also cortical structures with variable numbers of layers, principally the olfactory cortex, entorhinal cortex, the subicular cortices, and the hippocampus. The embryonic source of these structures is distinct from the embryonic source of the neocortex, including the cingulate cortices (*1*).

Distinct functional centers are found within the limbic system. Olfactory researchers highlight the olfactory bulb and vomeronasal organ and their primary targets, the primary olfactory cortex (“pyriform cortex”), amygdala, other basal forebrain nuclei of the olfactory tubercle, and the direct and secondary targets of the hippocampus and thalamus (*2*, *3*). Memory and spatial navigation researchers concentrate on the details and subdivisions of the hippocampus, entorhinal cortex, and other immediately adjacent cortical structures (*4*, *5*). Basal forebrain nuclei, principally the septum and amygdala, are central to the discussions of the subcortical “emotional brain” (*6*, *7*).

Even with these disparate functions, integration over these nuclei and cortices is routinely emphasized. For example, “Papez’s circuit,” a collection of tracts often featured in medical textbooks, is so prominent that it can be traced by blunt dissection, linking the hippocampus via the fornix to the hypothalamus, then to the anterior nucleus of the thalamus, to limbic cortices, and back to hippocampus. It is the rare current neuroscience textbook that omits the special emotional salience of olfactory memories. Unfortunately, one popular account that integrates over these diverse regions, the “triune brain” theory (*8*), persists to this day in the evocation of “my lizard brain” to explain uncontrolled emotional excess using the utterly false evolutionary tale that reptiles have brain only “up to” the emotional limbic system, grown over later by the logical neocortex, reaching its full flower in humans.

Fortunately, theories much better integrated over evolutionary, embryonic, neurophysiological, and neurological evidence have been developed. For example, the whole brain has been characterized by two ascending systems: (i) an ascending core concerned with evaluative sensation and physiological maintenance, integrated at the level of the hypothalamus and limbic regions, and (ii) a fast-conducting analytical pathway (as in rapid skin sensation, vision, and hearing), organized at the level of the thalamus and neocortex (*9*). The brain becomes integrated over these two systems for movement selection and affect in the basal ganglia of the forebrain. Another view ranks cortical structures across both limbic and neocortical origins in a hierarchy of prediction and control of emotional, motor, and sensory states, choices, and evaluation (*10*). A characterization of the forebrain based on developmental gene expression gives a better original segmentation and characterization of changing segmental and neuronal fates in the limbic and neocortical forebrain as brains enlarge (*1*).

The need for any complementary characterization of forebrain at all may be called into question. We raise a few of the basic issues in the neocortex-limbic system distinction: Empirical examination of whether an aroma, song, or vista better evokes an individual’s old memories is scarce; the hippocampus gets rather little olfactory input in many mammals and cannot be viewed as an olfactory structure; and the functions of the hippocampus and neocortex both span the domains of emotion, event, and sensorimotor world models. Yet, we argue here that the evolution and development of the vertebrate brain force a two-system view into renewed focus. This is not an invocation of a “new brain–on–old brain” layering along a scala naturae of fish, reptile, monkey, and human; rather, all vertebrates share essentially the same gross forebrain–to–spinal cord segmentation, with no segments added on. Within those segments, the general functional commitment of regions remains remarkably stable, with the anterior-most and lateral-most regions of the embryonic neural plate destined to proliferate relatively the most in larger brains (*11*).

Within this conserved organization, a strong pattern of systematic covariation between the cortical elements of the forebrain was first described quantitatively by Jerison (*12*) and was subsequently observed between all large vertebrate taxonomic groups that have been studied (*13*, *14*), within taxonomic groups (*15*), and in individual variation within species, including our own (*16*). Here, in the first section, we assemble the data from these multiple sources to highlight an inverse pattern of covariation between the limbic system and the neocortex, together with positive covariation among limbic system components. We argue that this two-system organization is characterized by distinct connectivity patterns in the component networks. The olfactory system and hippocampus have, as their base, a widely distributed pattern of axon extension, whereby distributed neural activity patterns identify a particular odorant in the case of the olfactory structures (*2*) and a unique event or location in the case of the hippocampus (*4*). The neocortex, in contrast, preserves local spatial relationships in the projections of its subregions, cortical areas, onto each other. In most of the lateral convexity of the cortex, the dimensions that are mapped between areas represent egocentric space, over several sensory modalities and the motor modality, with cortical maps maintaining nearest neighbor relationships of the sensory and motor surfaces (*17*–*19*). In part of the cortex (the temporal lobe in humans), spatially ordered cortical projections are used for nonegocentric mapping, as in tonotopy in auditory cortical representations or the often hybrid representations of faces, scenes, words, and concepts (*14*, *20*).

Next, we hypothesize that the reason for these different patterns of axonal projections in the two systems is a computational one: that optimal representations of the associated modalities are achieved with distinct network connectivity patterns that embed system-specific inductive biases. To test this, we encode the representations of high-performing artificial neural networks in two-dimensional model cortical sheets using topology-preserving maps. We find that networks optimized for visual, tactile, and auditory representations converge to orderly spatiotopic maps that maintain nearest neighbor relationships of the sensory input, resembling sensory maps and connectivity profiles in the neocortex. In contrast, networks optimized for olfactory and relational memory representations develop fractured maps with distributed connectivity profiles resembling axon distribution patterns of the olfactory-limbic system.

We then examine whether these differences in network connectivity can, in principle, explain macroevolutionary patterns of covariation among brain components. We use an evolutionary algorithm to optimize a multimodal network consisting of two distinct connectivity domains—one spatiotopic and the other distributed—each comprising multiple subnetworks. Selecting for different task objectives, we find patterns of covariation among subnetworks that resemble the negative covariation between the limbic system and the neocortex and the positive covariation among limbic system components observed across species. Together, these results suggest that the observed patterns of covariation between brain components arises as a consequence of a basic computational duality in brain evolution.

## RESULTS

### Neocortex and limbic system covariation

**Fig. 1.**
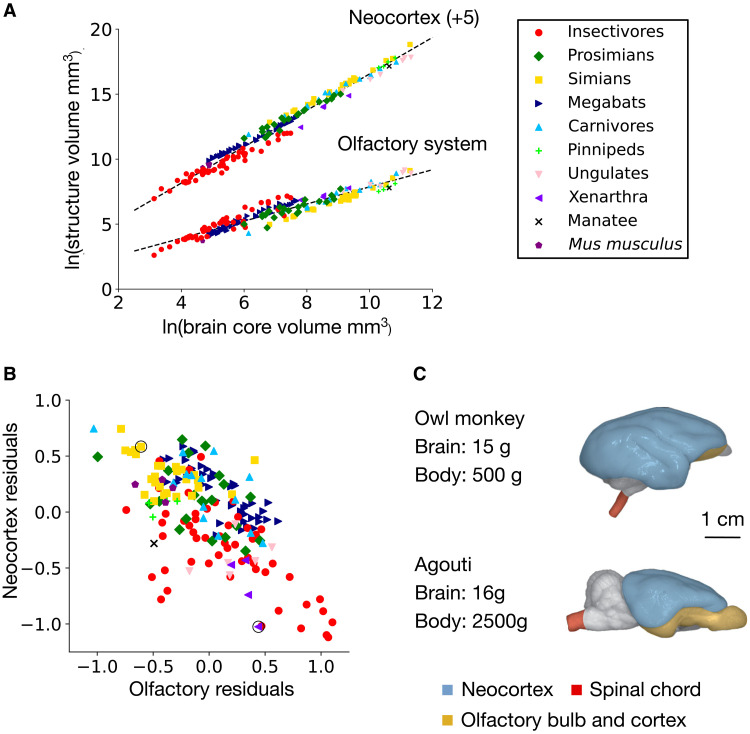
Inverse relationship between the olfactory system and the neocortex. (**A**) Volume of the olfactory system and neocortex plotted against the volume of a core set of brain components (medulla, mesencephalon, diencephalon, and striatum) for 182 mammalian species across 10 taxonomic groups. The olfactory system shown is the combined volume of olfactory bulb, olfactory cortex, olfactory tubercle including other medial forebrain nuclei, and the amygdala. The neocortex volumes are shifted by a constant (+5) on the *y* axis to separate the plots visually. Both structures are strongly correlated with brain core volume (Pearson *r* = 0.95, *P* < 0.001 for the olfactory system; *r* = 0.99, *P* < 0.001 for the neocortex). The regression line running through each set of scatter points has slope β = 0.66 for the olfactory system [hypoallometric; 95% confidence interval (CI): 0.63 to 0.69], and β = 1.40 for the neocortex (hyperallometric; 95% CI: 1.37 to 1.43). (**B**) Residuals of the olfactory system and neocortex around their corresponding regression lines, showing negative covariation between the volumes of the two structures (*r* = −0.63, *P* < 0.001). Because of the log axis in (A), an axis unit in (B) corresponds to a factor of *e* difference between expected and actual volumes. The armadillo (*Dasypus novemcinctus* from the Xenarthra taxonomic group) and the squirrel monkey (*Saimiri sciureus* from the Simian taxonomic group) discussed in the text are circled. (**C**) Lateral view of the brains of an owl monkey (*Aotus azarae*, a small nocturnal primate) and an agouti (*Dasiprocta agouti*, a large diurnal/nocturnal rodent), chosen to demonstrate the strong contrast in the relative size of the olfactory system versus the neocortex in brains of similar overall volume. The panel is adapted with permission from (*14*).

We use data collected for analysis of brain structures in 182 mammalian species across 10 taxonomic groups (*15*–*16*, *21*–*23*). Figure 1A plots the sizes (volumes) of the olfactory system and neocortex against the size of a core set of brain components (medulla, mesencephalon, diencephalon, and striatum). As the core components enlarge, both the olfactory system and neocortex scale predictably at characteristic rates, exemplifying the principle of concerted scaling, in which diverse brain regions expand together in a coordinated, developmentally constrained manner as the overall brain size increases (*24*).

The regression line running through each set of scatter points provides the allometrically expected volume of each structure, given the volume of the core brain components. We measure the deviations from this allometric expectation by calculating the residuals of the scatter points around their corresponding regression line. Plotting these residuals against one another reveals a notable inverse relationship between the two structures (Fig. 1B). Higher olfactory system residuals are associated with lower neocortex residuals, and vice versa, indicating that expansion of one structure above allometric expectations is accompanied by reduction of the other. This inverse relationship can be partly understood by the dominant sensory systems of the species involved. For example, the nine-banded armadillo (circled Xenarthra in Fig. 1B) is primarily nocturnal, relies on olfactory cues for foraging, and has a relatively large olfactory bulb and olfactory cortex (*25*, *26*). In contrast, the squirrel monkey (circled Simian in Fig. 1B) and other primates are highly visual, with a large fraction of the neocortex taken up by primary and extrastriate visual cortices (*27*, *28*). This contrast is further illustrated in Fig. 1C, which compares lateral views of the brains of an owl monkey and an agouti. The brains are similar in volume, but the difference in the relative sizes of the olfactory system and neocortex is apparent, with the neocortex in the primate obscuring both the olfactory system and cerebellum from view.

**Fig. 2.**
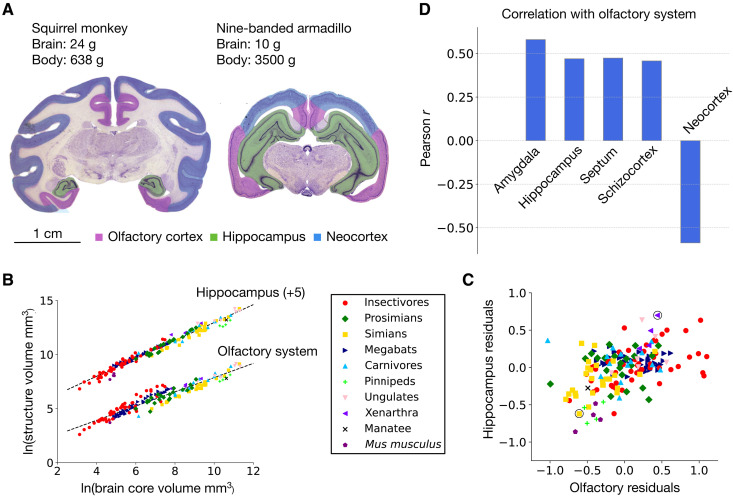
Covariation between limbic system components. (**A**) Cross section of the brain of a squirrel monkey (*S. sciureus*) and a nine-banded armadillo (*D. novemcinctus*) showing the relatively enlarged neocortex in the monkey (blue) and the relatively enlarged olfactory cortex (dark purple) in the armadillo. Note also the relative expansion of the hippocampus in the armadillo (green). (**B**) Volume of the olfactory system and hippocampus plotted against the volume of brain core. The hippocampus values are shifted by a constant (+5) on the *y* axis to separate the plots visually. Both structures are strongly correlated with brain core (Pearson *r* = 0.99, *P* < 0.001 for the hippocampus; *r* = 0.95, *P* < 0.001 for the olfactory system) and scale hypoallometrically. The allometric slope for the hippocampus is β = 0.83 (95% CI: 0.81 to 0.85), and that for the olfactory system is β = 0.66 (95% CI: 0.63 to 0.69). (**C**) Residuals of the two structures around their corresponding regression lines, showing positive covariation of their volumes (*r* = 0.46, *P* < 0.001). This is in contrast to the negative covariation between the olfactory system and the neocortex in Fig. 1B. The armadillo and squirrel monkey discussed in the text are circled. (**D**) The volumes of all limbic system components positively covary with the volume of the olfactory system. Shown are the Pearson correlation coefficients between the normalized volumes (residuals) of each structure and that of the olfactory system across species. The correlation coefficient of the neocortex is also shown for comparison (all *P* < 0.001). “Schizocortex” includes the entorhinal, perirhinal, presubicular, and parasubicular cortices. Amygdala volumes were available separately from the olfactory system for 102 of the 182 species analyzed. Images in (A) are from the Comparative Mammalian Brain Collections, www.brainmuseum.org, property of the University of Wisconsin and Michigan State Comparative Mammalian Brain Collections, funded by the National Science Foundation and the National Institutes of Health.

Figure 2A depicts a coronal section of the forebrain at the posterior thalamus level in a squirrel monkey and a nine-banded armadillo. The relatively larger olfactory cortex in the armadillo might be expected from its relatively greater reliance on olfaction, but its massive hippocampus is notable. In our dataset, the hippocampus in the armadillo is two times larger than its allometrically expected value, corresponding to 464 mm^3^ of expanded circuitry. In comparison, compared to allometric expectations, the olfactory system is 1.5 times larger, whereas the neocortex is reduced by a factor of 2.8. Across taxonomic groups, the hippocampus positively covaries with the olfactory system (Fig. 2, B and C), which contrasts with the negative covariation between the olfactory system and the neocortex described above (Fig. 1B). Why does the size of the hippocampus positively covary with that of the olfactory system? Although olfactory input to the hippocampus is strong, it is not dominating. The latter integrates multiple streams of information, including those originating in the neocortex, and thus is not a secondary olfactory structure (*4*, *29*, *30*). Instead, we argue in the following sections that the covariation between the olfactory system and the hippocampus reflects a common computational architecture, characterized by a common pattern of network connectivity, distinct from that of the neocortex.

In addition to the hippocampus, all other limbic system components, including the amygdala, septum, and schizocortex, positively covary with the olfactory system (Fig. 2D) and with each other. The covariation with the amygdala is the highest given the direct projections of olfactory cortex to that structure, but the covariations with the other limbic system components are also notable and significant. This concerted change of all limbic system components, together with their inverse covariation with the neocortex (fig. S1), suggests a two-system view of the macroscale organization of the brain. In early neurodevelopment, the two regions can be easily distinguished by their patterns of cell proliferation and early gene expression (*1*) and in the expression of transcription factors, notably LAMP (“limbic-associated marker protein”) (*31*), suggesting distinct developmental rules of network formation.

### Information structure and optimal representations

To develop a normative explanation for the patterns of covariation in brain evolution discussed above, we investigate the structure of distinct information sources driving brain computation and their optimal representations. For this purpose, we assess the representations of high-performing artificial neural networks that are optimized for one of four sensory modalities—vision, audition, somatosensation, and olfaction—followed by a network optimized for relational memory. The sensory systems consist of the following neural network architectures pretrained on large data corpora: (i) ResNet trained on images from the ImageNet database (*32*, *33*), (ii) wav2vec 2.0 trained on natural speech from the LibriSpeech corpus (*34*, *35*), (iii) a convolutional architecture trained on a dataset of tactile signals of the human grasp (*36*), and (iv) an olfactory system model trained on simulated olfactory receptor neuron (ORN) activations (*37*). These networks show close correspondence to neural activity and structural connectivity patterns in the brain within particular hyperparameter settings (*33*, *35*, *37*). A combination of network design and gradient-based training optimizes the parameters (connection weights) in each network, and consequently, the networks embed manifolds of stimulus feature information in their activity.

**Fig. 3.**
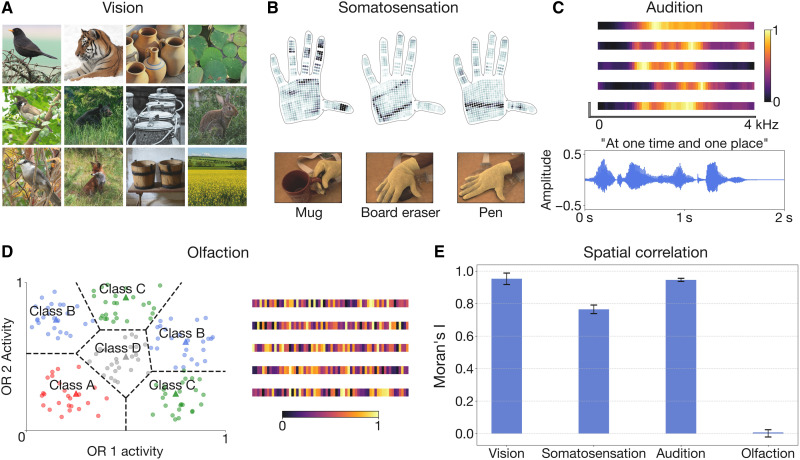
Information sources of four sensory modalities. (**A**) Samples from the ImageNet dataset. ImageNet images are characterized by spatial correlation visible in the form of contrastive backgrounds, repeating textures, and distinct natural shapes. (**B**) Top: Visualizations of pressure readouts from the tactile glove dataset . Bottom: Perspective images showing the creation of each sample. Activated regions of the tactile glove are highly localized, as clusters of adjacent sensors respond to pointwise contact with the object being classified. (**C**) One-dimensional cochleagram slices (top) and time-amplitude representation of a single sample (bottom), each from the LibriSpeech ASR corpus. In the cochleagram slices, frequency bands associated with human speech form localized patterns. (**D**) Left: Subset of 150 synthetic odors from the Wang *et al.* dataset showing activation values of two olfactory receptor neurons (ORNs). Each odor sample is encoded by 50 ORNs in the dataset. Individual odors are classified by their proximity to two prototype odors (triangles). Right: ORN activation profiles of five sample odors from the dataset. Neighboring ORNs exhibit no local spatial correlations. (**E**) Bar plot showing spatial autocorrelation (Moran’s I) across the aforementioned datasets. Moran’s I is the ratio of the spatially weighted covariance to the overall variance, normalized by the number of observations and the sum of the spatial weights. This metric is expressed in a range from −1 to 1, where positive values indicate increasingly strong local similarity, values near zero suggest random spatial patterns, and negative values indicate strong local dissimilarity.

The stimuli that each model is optimized for are depicted in Fig. 3 (A to D). We observe a dichotomy in the structure of information across modalities, with visual, somatosensory, and auditory data showing a pronounced spatial structure, as measured by spatial autocorrelation [Moran’s I (*38*)], and olfactory data lacking any such structure (Fig. 3E). Although coarse-scale chemotopy in olfactory receptor neuron population representations has been proposed in some studies (*39*), it is clear that they lack the fine-grained spatial order characteristic of primary visual, somatosensory, and auditory representations (*40*–*42*). We argue that this dichotomy in the structure of information sources underlies the two-system architecture that we propose, each system preconfigured with the knowledge of the core statistical structure of its input.

### Spatiotopic and disordered maps

After optimization of network parameters in the models, network activity constitutes an optimized population code of stimulus features across a multidimensional feature space. In this section, we hypothesize that spatially embedding these representations will reveal differences in their underlying organization.

A prevailing theoretical account of cortical organization posits that sensory feature spaces are represented in two-dimensional cortical surfaces in a manner that preserves the topology of the stimulus manifold (*43*–*47*). Similar stimulus features are represented in nearby locations on the cortical surface to aid efficient similarity-dependent computations. Following this account, we encode the feature representations of our models on a two-dimensional cortex-like surface using Kohonen self-organizing maps (SOMs) (*46*, *47*). We consider the organization of primary sensory maps that are in place early in brain development and whose activity drives areal organization across the system (*17*, *19*, *48*, *49*). Specific layers of our models show the strongest correspondence to activity in primary sensory areas (*33*, *35*, *37*). We construct SOMs using activity in these layers (Materials and Methods).

**Fig. 4.**
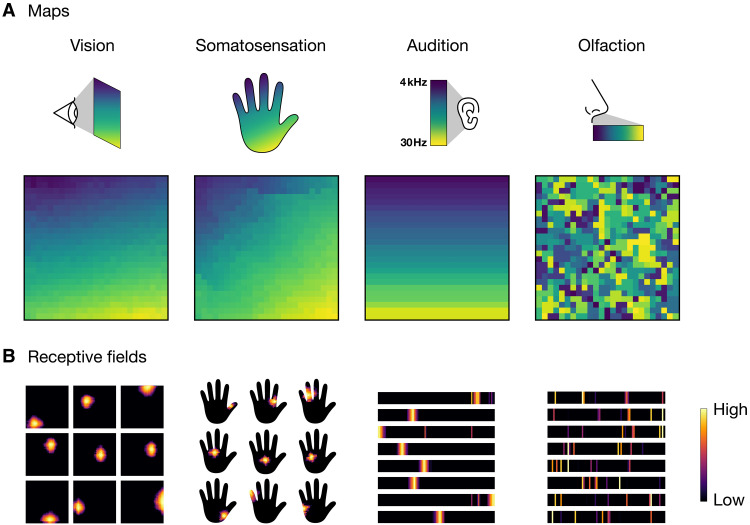
Spatial embedding of sensory systems. (**A**) Model cortical sheets constructed by encoding the representations of task-optimized artificial neural networks in two-dimensional sheets using topology-preserving maps. Models of four sensory systems are constructed. The top row depicts the sensory surfaces—two-dimensional physical space for vision and somatosensation, one-dimensional physical arrangement of frequency channels for audition, ordered by frequency, and one-dimensional physical arrangement of olfactory sensory neuron activations for olfaction. The sensory surfaces are color coded according to physical location. Neighboring locations on these surfaces have correlated activations in vision, somatosensation, and audition but not olfaction (Fig. 3). The bottom row shows maps of the sensory surfaces on two-dimensional model cortical sheets. Each sheet consists of 25 by 25 units. Each unit is colored according to the input location that maximally activates the unit. The gradual transition of colors in visual, somatosensory, and auditory representations indicate a smooth mapping of the input space on the cortical surface. No such mappings are found for olfactory representations. (**B**) Example receptive fields of model units indicating the degree to which each region of the input activates a unit. Receptive fields in vision, somatosensation, and audition are spatially localized, whereas those in olfaction are spatially distributed.

Figure 4A shows the maps that develop in each modality, highlighting two contrasting structures. Visual, somatosensory, and auditory maps converge to orderly representations that maintain nearest neighbor relationships of the sensory surface resembling spatiotopic organization that characterizes the neocortex. Olfactory maps, in contrast, develop fractured representations akin to disordered chemotopy in the olfactory bulb and cortex. We quantify the degree of global spatial order in each map by computing the mean Euclidean distance between each unit’s preferred stimulus and that of its adjacent units, normalized by the expected distance in a random arrangement and negated to result in an order metric (Materials and Methods). This results in an index that ranges from 0 (low order) to 1 (high order). We measure values of 0.90, 0.86, and 0.89 for visual, somatosensory, and auditory maps, respectively, and 0.11 for olfactory maps.

The visual, somatosensory, and auditory maps of Fig. 4A are constructed using the representations of their respective network models. These models are initialized with localized patterns of network connectivity, which is known to substantially improve task performance and training efficiency in these modalities (*50*–*52*). In contrast, the olfaction model is initialized without prespecified network structure (*37*) and instead develops sparse, distributed connections over the course of gradient-based learning of olfactory signal representations. Imposing localized network connectivity in this model before learning leads to a sharp decrease in olfactory task performance and generates maps that remain fractured and disordered (fig. S2). Maps generated by the network models before they are trained on their respective datasets are shown in fig. S3.

We next assess the receptive fields of individual SOM units and find that visual, somatosensory, and auditory units draw information from localized regions of the sensory surface, whereas olfactory units sample across distributed parts (Fig. 4B), recapitulating a central distinction between afferent projection patterns in the primary olfactory cortex and primary sensory areas of the neocortex. We measure the degree of locality of the receptive fields by computing the spatial autocorrelation (global Moran’s I) of representative SOM unit receptive fields, again resulting in an index that ranges from 0 (low locality) to 1(high locality). We calculate locality indices of 0.97, 0.84, and 0.99 for vision, somatosensation, and audition, respectively, and 0.00 for olfaction. To assess whether these differences depend on the specific choice of network architecture, we additionally train fully connected networks from scratch on each modality (*53*). Even in the absence of architectural priors, networks trained on visual, somatosensory, and auditory data develop localized connections, whereas networks trained on olfactory data develop distributed connections (fig. S4). The contrasting structure of information representation between the spatiotopic and olfactory modalities mirrors the dual structure of their information sources (Fig. 3) and underscores a pronounced duality in the organization of optimal sensory representations.

### Relational memory

**Fig. 5.**
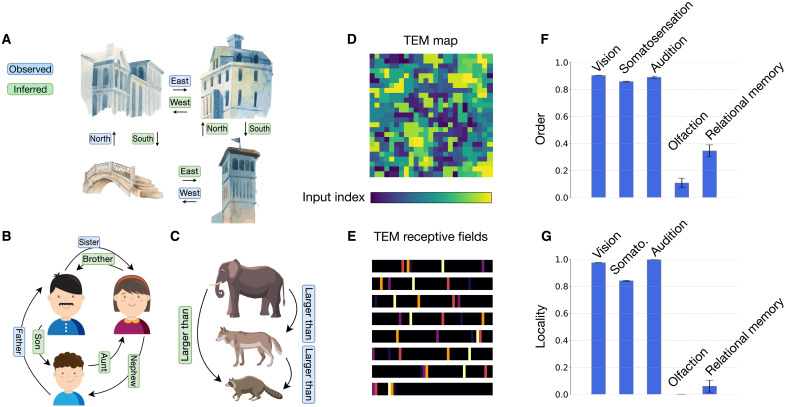
Spatial embedding of relational memory. The TEM learns the relational structure of a problem and uses that knowledge to make quick inferences in new problem instances . Relational structure exists in many situations, for example, (**A**) spatial reasoning, (**B**) social hierarchies, and (**C**) transitive inference. The TEM infers the green relationships after observing only the blue ones using a generative model of the hippocampus and entorhinal cortex. Clipart designed by Freepik. (**D**) The hippocampal activity of the TEM is encoded in a two-dimensional surface using SOMs. Shown is a surface consisting of 25 by 25 units, where each unit is colored according to the location of the input that maximally activates that unit. The disordered map resembles the olfactory map of Fig. 4A. (**E**) Example receptive fields of hippocampal units in the TEM. The receptive field of a unit (one-dimensional horizontal bar) shows the degree to which each region of the input activates that unit. The spatially dispersed receptive fields resemble those of the olfactory map in Fig. 4B. (**F** and **G**) Indices measuring the order of the maps and the locality of the receptive fields across the five modalities. Hippocampal relational memory and olfactory representations have distinct organization compared to the spatiotopic sensory systems as indicated by the values of these indices. Plotted are the means and standard deviations across 10 simulations with different random seeds.

Next, we assess representations in the Tolman-Eichenbaum Machine (TEM), an artificial neural network for relational inferences modeled on computations in the hippocampus and entorhinal cortex (*54*). The network is trained to learn conjunctions of perceptual and structural representations and develops a cognitive map via which relationships between objects and their spatial or abstract locations are inferred (Fig. 5A). Activity within the network resembles activity patterns in spatial and nonspatial cell types in the hippocampus and entorhinal cortex, as well as their structural remapping under new stimuli (*54*). We encode the conjunctive representations of this model in a two-dimensional surface using a SOM to compare its structural features to the sensory systems maps of Fig. 4.

As in the olfactory maps, hippocampal maps develop fractured representations that lack global spatial order, with SOM units drawing information from distributed parts of the TEM’s input (Fig. 5, D and E), resembling sparse and distributed coding in the hippocampus (*5*, *55*, *56*). We compare map indices across models and observe that both olfactory and hippocampal representations exhibit low spatiotopic order and distributed receptive fields, indicating that the associated computations require a network architecture that is distinct from those of the other sensory systems (Fig. 5, F and G). These results hold across different settings of the SOM algorithm (figs. S5 and S6). Imposing local connectivity within the hippocampus component of the TEM before learning generates a map that remains spatially disordered and results in a substantial degradation in generalization performance, as also observed for olfaction (fig. S2). The connectivity structure essential for high task performance in the spatiotopic modalities is thus detrimental to olfactory and hippocampal computations.

The lack of spatial topography in TEM representations when mapped to a two-dimensional surface is consistent with experimental evidence that episodic memory in the hippocampus is encoded by highly distributed, sparse population codes rather than smooth maps. Recent work in food-caching chickadees shows that each caching event evokes a unique, transient “barcode” pattern of population activity that is uncorrelated even between adjacent sites and that these barcodes reliably reactivate during the retrieval of the same cache (*5*). Similar results have been reported in rodents and humans, where hippocampal ensembles display event-specific reactivation during recall without preserving spatial adjacency, supporting the idea of a high-dimensional “index” code rather than a continuous map (*57*, *58*). Our model supports this principle by generating a fractured, globally distributed map that nonmetrically tiles its representational space.

### Evolution of a dual computational system

The network connectivity patterns discussed so far resemble the macroscale architectures of primary sensory systems in the brain, which are established early in development (*17*, *48*). This preconfiguration of network structure before stimulus exposure serves subsequent computations by encoding knowledge of the structure of expected stimuli in network connectivity. What developmental factors shape this connectivity and what axes of variations are offered up to natural selection? Here, we examine whether a single dimension of variation could explain the negative covariation between the limbic system and the neocortex and the positive covariation among limbic system components observed across species (Figs. 1B and 2C and fig. S1b).

**Fig. 6.**
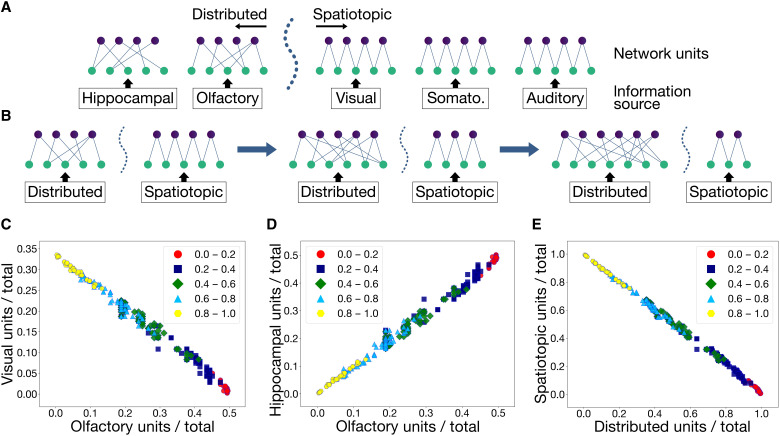
Patterns of covariation generated by an evolutionary algorithm. (**A**) A dotted boundary separates two subnetworks with distributed connections from three subnetworks with spatiotopic (local) connections. Shifting this boundary adjusts the number of units allocated to each side. Each generation consists of multiple networks, with the boundary positioned differently in each network. New generations of networks are produced by selecting the fittest networks of the previous generation and shifting the boundary in a direction (left or right) chosen at random. (**B**) Depiction of selecting for olfaction. The boundary shifts in a direction that expands the distributed domain, resulting in a larger allocation of units to the subnetworks in that domain. (**C**) Allocations to the visual and olfactory subnetworks after 50 iterations of variation and selection. Points are categorized by groupings of values of the parameter *s*, which ranges from 0 to 1. This parameter is the weight assigned to the test performance of vision when calculating the fitness of a given network. A high value of *s* selects for vision, and a low value selects for olfaction. (**D**) Similar to (C) but showing allocations to the hippocampal and olfactory subnetworks. Note that the hippocampus is not a direct target of selection but instead expands or contracts with the spatial extent of distributed connectivity. (**E**) Plot showing the combined allocations to the visual, somatosensory, and auditory subnetworks (*y* axis) against the combined allocations to the olfactory and hippocampal subnetworks (*x* axis) after 50 iterations.

We configure a dual-system architecture composed of two broad domains of axon extension patterns: one spatiotopic and the other distributed. Each domain consists of modality-specific subnetworks. The spatiotopic domain includes subnetworks for vision, somatosensation, and audition, while the distributed domain consists of subnetworks for olfaction and hippocampal relational memory (Fig. 6A). Network units within each domain are allocated equally among that domain’s subnetworks. This setup is motivated by our results in the previous sections that demonstrate that distributed connectivity is optimal for olfactory-limbic processing, whereas spatiotopic connectivity is optimal for vision, audition, and somatosensation in our models. We ask how these networks may covary under selection pressures along a single dimension of variation—the location of the boundary dividing the spatiotopic and distributed domains. The boundary determines the number of units, or the amount of computational resources, allocated to each domain, and its variation accounts for a minimal degree of stable developmental control of tissue allocation (Fig. 6B and fig. S7).

We use an evolutionary algorithm (*59*, *60*) to optimize the position of the boundary under a selection criterion defined by a parameter *s* that takes a value between 0 and 1; a low value of *s* selects for olfaction, and a high value selects for vision. We initialize a population of networks, each with an approximately even allocation of units between its subnetworks, and run the evolutionary algorithm for 50 optimization steps. At each step, the networks with the highest task performance are selected, and the locations of their boundaries are varied uniformly at random to generate networks for the subsequent step (Materials and Methods). Task performance is a combination of olfactory and visual signal identification, as defined by the parameter *s*. The algorithm optimizes the performance of the networks across iterative steps of variation and selection.

Figure 6C shows the outcome of the evolutionary algorithm for different values of *s*. When olfactory performance is selected for (low values of *s*), the number of units allocated to olfaction increases at the expense of vision, and vice versa (high values of *s*), recounting the olfaction-versus-vision account of sensory specialization (Fig. 1, B and C). Notably, instead of singular expansion of the selected component, a concerted change in the size of all components takes place, with the olfactory and hippocampal subnetwork sizes positively covarying with one another and inversely covarying with the combined size of the spatiotopic subnetworks (Fig. 6, D and E). The hippocampus model is not a direct target of selection but rather expands or contracts as a consequence of its architectural similarity to the olfactory subnetwork. Scaling trends are independent of the particular settings of the evolutionary algorithm, with the same overall variations observed for a wide range of settings (figs. S8 to S10). These results indicate that, in principle, a minimal degree of developmental control—specifying the spatial extent of two distinct patterns of axon outgrowth—can account for macroscale covariation patterns among brain components. A segmental “prosomeric” structure of the forebrain defined by domains of regulatory gene expression suggests a mechanism of this nature (*1*, *61*, *62*).

When evolution selects for brain components, it selects for the function that arises from underlying computational processes. Particular computations are aided by specific features of network architectures and representational schemes, the spatiotopic and distributed networks discussed here being examples from what may be an abundant space of architectural priors exploited in brain computation (*63*, *64*). Variations in network organization offered up to natural selection are shaped by developmental processes and thus constrained by directions of variation under stable developmental control. These “units of development” might not precisely correspond to the function that is selected for, and the outcome of selection is likely a result of specific functional demands and available developmental variation, mediated strongly by metabolic costs and other contingencies (*15*, *18*, *65*). The results presented here suggest that the pattern of covariation between the limbic system and the neocortex observed across species is a consequence of developmental control of tissue allocation between two computational systems offered as loci of selection. Despite the diversity of limbic system function, a common computational architecture and coarse developmental structure underlie concerted variation of its components. Selecting for one causes the selection of all, with pleiotropic effects on behavior.

## DISCUSSION

The results we report here suggest that the mammalian forebrain is shaped by a trade-off between two computationally distinct systems, one favoring spatiotopically organized representations and the other supporting distributed coding. We combine comparative neuroanatomical data with normative models of information representation to argue that neocortical and olfactory-limbic components lie on opposite poles of a coordinated developmental and functional axis, with expansion of one system accompanied by contraction of the other, once concerted allometric effects are accounted for. Rather than being an incidental consequence of brain scaling, this inverse relationship appears to reflect an allocation of computational resources on the basis of the statistical structure of sensory inputs and functional demands. From this perspective, we interpret macroscopic evolution of forebrain components as a series of adjustments along a single axis of variation, balancing spatially organized computations with sparse distributed memory.

Our arguments are based on the premise that wiring rules during development encode knowledge of the core statistical structure of sensory scenes within brain network architectures before experience-dependent learning. Developmental processes that establish the connectivity of primary visual, somatosensory, and auditory cortices have been characterized by decades of experimental work on the molecular mechanisms that attract, sort, and spatiotopically organize input from their respective thalamic nuclei (*66*). Associated theoretical arguments and models of network formation suggest that endogenous activity within these primary cortical areas establish a series of topographically organized higher cortical maps, subsequently refined by experience-dependent learning (*17*, *19*, *49*). To what extent the distributed connectivity patterns of olfactory-limbic structures are specified by development or shaped by learning is known to a lesser degree. Developmental precision of olfactory sensory neuron convergence patterns onto glomeruli by a hierarchy of axon guidance cues indicates strong preconfiguration of olfactory circuit architectures before learning (*41*). However, the degree of developmental structure imposed on the distributed, seemingly random, projections from the olfactory bulb to the cortex, central to the acquisition of olfactory memories, remains an open question. In the hippocampus, distinct developmental phases coalesce preconfigured and experience-dependent cell assembly sequences during the formation and consolidation of episodic memories (*67*). In a similar expression of mixed developmental and learned structure, stereotyped circuits in the amygdala that drive innate responses are additionally recruited during reward-modulated learning (*68*). These observations support our premise that developmental processes encode inductive biases through early wiring rules, providing a scaffold for representations subsequently acquired through learning.

Normative accounts of brain network organization postulate that connectivity features of neuronal networks emerge as a consequence of optimization for particular functional objectives (*69*–*71*). Advances in artificial neural network design and training procedures over the past decade have led to a number of such accounts linking connectivity and response properties of model networks to empirical data. For example, receptive fields in the primate ventral visual system resemble those of network units in deep convolutional networks (*33*, *46*, *72*, *73*), such as the ResNet architecture we examine here. These properties arise in the models after training on large natural image datasets using task-dependent or task-agnostic objectives and gradient-based learning rules. Aspects of mesoscopic functional organization in the brain also emerge in the models once physical constraints of two-dimensional surface representations are taken into account. Similar results have been reported for auditory and language regions (*35*, *74*). Although the training procedures of these models bear little resemblance to training and optimization processes in the brain, the observed correspondence between their tuning profiles suggests that it is the structure of the stimulus space rather than the particularities of the optimization process that determines network organization and response. These models, however, should not be overinterpreted, given the limited explanatory power of today’s artificial neural networks and their indirect relationships to representations and mechanistic processes in the brain (*75*).

Prior work linking representations of deep neural networks to spatial organization in the cortex has taken two complementary approaches. Topographic deep artificial neural network architectures (*33*), including related connectivity-constrained models (*72*), induce smooth spatial maps on a two-dimensional surface by incorporating explicit wiring-cost constraints into the training objective, resulting in representations that quantitatively fit empirical data. An alternative approach, which we adopt here, is the use of Kohonen SOMs as an analysis tool applied after training to embed and visualize learned representations (*46*, *47*). While these approaches differ algorithmically, they both impose spatial structure by encouraging nearby neurons to develop correlated responses, and they both address the question of whether network representations can be smoothly embedded on a model cortical surface.

Our analysis addresses one major axis of forebrain organization, but several limitations should be considered. First, the neuroanatomical datasets we used were aggregated from multiple sources, which, although extensive, are subject to measurement variability and limited sampling in some taxonomic groups. Future work with higher-resolution datasets—such as those generated by automated segmentation of whole-brain imaging—could refine these allometric relationships. Second, cortical topographies are complex and multidimensional, comprising multiple overlapping feature maps and tuning gradients that vary across areas and species (*19*, *76*). Our analysis abstracts away this fine-grained structure to highlight a macroscopic feature shaping brain evolution and therefore does not aim to provide a detailed account of cortical map organization. Also, our models do not incorporate mechanistic detail, relying on simplified unit responses and network interactions. The internal representations of the models are therefore not direct analogs of cortical activity. Rather, we use our models as idealized tools to identify features of network connectivity and spatial organization that are favored by different classes of information under optimization for functional objectives.

Last, the evolutionary model we use is intentionally minimal in its formulation. Motivated by developmental compartmentalization of the forebrain, it represents resource allocation as a single boundary parameter between two competing domains, setting aside effects of graded gene-expression boundaries, developmental timing, and nonlinear growth. Extending this model to incorporate these factors would yield a richer testbed. These simplifications mean that the results of the evolutionary algorithm are best viewed as demonstrating the principle that a small number of developmental control parameters can explain large-scale covariation patterns, rather than as a process-level description of evolutionary change.

The overall volumetric pattern of mammalian brain evolution is astonishingly coordinated and positively correlated part to part (*24*), so the negative covariation between the limbic system and the neocortex that we highlight here suggests a fundamental divergence in computational strategies for processing distributed versus spatiotopically organized information. A similar “inverse resource allocation” between smell and vision has been observed across multiple species of *Drosophila*, so the computational problem to be solved may be applicable outside of a single vertebrate line (*77*). If a hippocampus is unusually large in a taxonomic group, does it take over comparatively more functions or develop hierarchy or regionalization in some parallel fashion to the neocortex? During brain development, little is known about how the various levels of complexity are nested or evolvable, from the branching patterns of single neurons to the distributed or specific connectivity within individual circuits, to the network connectivity of multiple structures. So far, most of the empirical information that feeds network-level analysis comes from studies of “laid-out” structures, like the lateral convexity of the neocortex, the olfactory bulb, or the hippocampus. Still, the nuclear structures communicating with these regions appear to have connectivity rules congruent with their targets, comparing the precise connectivity of thalamocortical connections, for example, to the distributed output of the amygdala. The covariation of the volumes of the nuclear components of the limbic system we point out here suggests a shared developmental instructional system independent of layering, one that has scarcely been studied.

## MATERIALS AND METHODS

### Neuroanatomical data

Brain volume measurements of insectivores, prosimians, and primates are taken from (*21*, *22*); macrobats from (*23*); carnivores, pinnipeds, ungulates, xenarthra, and the manatee from (*15*); and *Mus musculus* from (*16*). Together, these cover a wide range of mammalian species, a diverse array of niches (ranging from burrowing to flying, nocturnal and diurnal, omnivores and specialists), and a wide spectrum of brain sizes. Volumes are listed for a core set of brain components—medulla, mesencephalon, diencephalon, and striatum—as well as limbic system components, neocortex and cerebellum. Limbic system components are amygdala, olfactory system, septum, hippocampus, and entorhinal cortex and associated cortical structures (parahippocampal, presubicular, parasubicular, and subicular cortices). Structure nomenclature and phylogenetic assignments are matched to the initial work of Stephan and colleagues (*21*, *22*). We omitted an outlier insectivore species (*Geogale aurita*), which has an unusually small neocortex, as discussed in (*21*). An analysis incorporating phylogenetic corrections for shared evolutionary history among species is presented in (*15*).

Following Stephan *et al*. (*22*) and Reep *et al*. (*15*), the olfactory system volume in Figs. 1 and 2 is the combined volume of olfactory bulb, olfactory cortex, olfactory tubercle including other medial forebrain nuclei, and the amygdala. See Reep *et al*. (*15*) for the minimal effects of inclusion or exclusion of the amygdala. Figure 1C (amygdala bar) considers the amygdala separately from the olfactory system in the 102 species for which the volumes are separately available.

Allometric relationships are estimated using ordinary least squares regression on log-transformed volumes ln(*V*_structure_) = α + β ln(*V*_core_), where *V*_structure_ denotes the volume of the brain structure of interest, and *V*_core_ denotes the volume of the core set of brain components. The slope and intercept of each structure examined are listed in table S1.

### Self-organizing maps

Kohonen SOMs are constructed using the activity of pretrained deep neural networks (*44*, *46*, *47*). The spatial organization of maps arising from five distinct networks is assessed, as described in the main text. The constructions of the maps are consistent across all the networks.

To construct each map, the corresponding network’s input space is divided into spatially nonoverlapping patches of input. The set of patches is passed through the network, one at a time, and activations arising from the final feature extraction layer (specified below) are recorded. This constitutes the set of activations that are used for constructing the SOM. This will be referred to as set *A* below.

The SOM is initialized with 625 units arranged in a 25 by 25 square lattice. Elements from set *A* are then presented to the SOM as input, one at a time. Each SOM unit computes a weighted sum of this input; the weights are chosen uniformly at random at initialization. The most active SOM unit and units within its local neighborhood are then selected, and their weights are updated such that the units develop similar receptive fields. This process is repeated for a total of 100,000 iterations through *A*. The map generation algorithm is shown in Algorithm 1 below. A hyperparameter sweep is provided in figs. S5 and S6.

**Algorithm 1.**
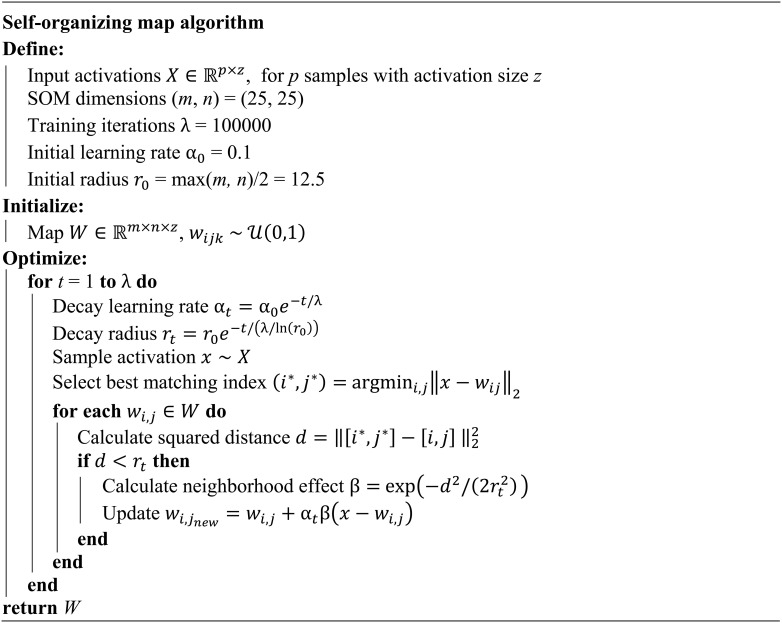
Self-organizing map algorithm.

To visualize the map, a smooth color gradient is set up to index all the patch locations that were used to construct *A*. After constructing the map using the algorithm described above, elements of *A* are again passed to the map, one at a time. The map unit that is most active for each element is assigned the corresponding patch’s color. The map is then visualized as a 25 by 25 heatmap, where each cell corresponds to a SOM unit and colored accordingly (Fig. 4A of the main text).

The location preference of a map unit is defined as the patch index for which that unit had its highest activation. The spatial order (smoothness) for each map (Fig. 5F of the main text) is quantified by examining the location preference of each map unit. For each unit, the average distance between its location preference and the location preferences of immediately adjacent units is calculated. This is done for all units in the map and averaged. This average distance is then divided by a normalization factor, representing the expected distance in a random map computed with 10,000 Monte Carlo samples. The resulting metric is then negated to quantify smoothness, and the range is normalized to 0 to 1.

The receptive field of a map unit is visualized as a heatmap whose dimensions match the shape of the input data (Fig. 4B of the main text). The intensity of each cell in the heatmap corresponds to the degree of activation of the map unit in response to input at that cell location. The top decile of activations is shown. The receptive field locality measure (Fig. 5G of the main text) is calculated by averaging the spatial autocorrelation (global Moran’s I \cite{moran1950notes}) of the receptive fields of all units in the map.

### Stimulus datasets

The vision map is constructed using activity from a ResNet18 model pretrained on the ImageNet dataset (*32*, *33*). The activations are recorded from layer 4 after the final CNN feature extraction block. The raw data input size is 225 by 225 by 3. The patch size is 3 by 3 by 3, with patch elements assigned values between 0.5 and 1, chosen uniformly at random, resulting in 5625 patches for constructing the map. Before presenting the dataset of patches to the network, the dataset is normalized to have the same mean as a subset of the ResNet18 training dataset.

The somatosensory map is constructed using a one-frame grasp classification network, trained on a dataset of tactile signals of the human grasp following the procedure outlined in (*36*). Activations are recorded from layer 10 after the ResNet feature extraction block, maintaining consistency with the vision network. The raw input data shape is 32 by 32 by 1. The patch size is 1 by 1, with values between 0.5 and 1, chosen uniformly at random, resulting in 1024 patches for constructing the map. The patches are filtered to only include patch coordinates with nonzero pressure values in the network’s training dataset.

The audition map is constructed using a wav2vec 2.0 model pretrained on natural speech from the LibriSpeech corpus (*34*, *35*). Activations are recorded from layer 7 after the feature projection layer of the encoder block, consistent with the somatosensory and vision networks. The raw waveform is truncated to 3000 frames and transformed to a spectrogram with 5000 frequency channels. Patches are created in the frequency domain, where each patch is a single frequency channel. Patch values are drawn uniformly at random from the range [0, 1] and then mapped to the range [11, 22], matching the distribution of the raw data. After patch construction, the data are transformed back to a waveform format. The SOM weight-update function is modified to accommodate the one-dimensional frequency data by only considering a single dimension distance in the update function.

The olfactory map is constructed using the olfactory system model in (*37*). The input size of the model is increased from 50 to 4096 to use the same SOM size as those in the other modalities. Activations are recorded after the third layer, corresponding to the pyriform cortex in the model. The patch size is 1 by 1, with values assigned between 0.5 and 1, uniformly at random. Before presenting the dataset of patches to the network, the dataset is normalized to have the same mean as the network’s training dataset.

The hippocampal map is constructed using activity in the TEM (*54*) with an observation dimension of 45. The network is probed using a single batch of 100 rollouts and 45 basis vectors (vectors of zeros with a single 1). At each step in the rollout, each basis vector is passed to the model, and the activation of the seventh inference layer, corresponding to the calculation of the inferred grounded location, is recorded.

### Evolutionary algorithm

The evolutionary algorithm operates on the parameter allocations of a neural network consisting of two domains, one with spatiotopic connections and the other with distributed connections. Each domain consists of multiple subnetworks—visual, somatosensory, and auditory subnetworks in the spatiotopic domain and olfactory and hippocampal subnetworks in the distributed domain. Each subnetwork has its own input, hidden, and output layers. The subnetworks are trained simultaneously in PyTorch using modality-specific datasets. Because of the computational requirements of evolutionary algorithms, smaller datasets are used for this assessment. These are CIFAR-10 for vision, Speech Commands for audition, and an associative memory dataset for the hippocampal subnetwork (*56*). The somatosensory and olfactory datasets are the same as those in the other assessments.

In the setup described above, each subnetwork in the spatiotopic domain has a hidden layer that draws information from localized 5 by 5 regions of the input. In contrast, each subnetwork in the distributed domain has a hidden layer that is fully connected to the input, with a random subset of weights set to zero. The size of this random subset is chosen such that the active parameters in the subnetwork are roughly equal to those of a locally connected subnetwork of the same size.

Hidden layer sizes in the subnetworks are determined by a boundary *B* between the spatiotopic and distributed domains. The position of this boundary [indicated by the dotted line in Fig. 6 (A and B)] determines the allocation of network units to each domain. Units allocated to a domain are distributed evenly among the domain’s subnetworks.

The evolutionary algorithm is run for 50 iterations, each with a population of 50 networks. The networks differed in the position of the boundary *B* and, thus, in the allocation of units among the subnetworks. First, a seed network is constructed in which the boundary is placed at a location that divides the total available parameters evenly across all five subnetworks. The initial population is constructed by sampling uniformly within ±10% of the seed network’s boundary location, with the seed network included by default in the first generation.

Next, the networks are trained with gradient descent for 50 epochs. The fitness of each network is measured using a weighted combination of task performance in the visual and olfactory modalities, described below. Twenty-five networks (half of the population) that have the highest fitness scores are then selected for the next generation, along with an additional 25 networks sampled with boundary values centered around that of the most fit network in the current generation. The results do not depend on particular choices of these settings; the same overall results are observed for a range of settings (figs. S8 to S10).

The fitness function weighs task performance using a parameter *s*, where a value of 1 fully emphasizes visual performance and a value of 0 fully emphasizes olfactory performance. The other modalities do not affect fitness in this setup. Varying the parameter *s* between 0 and 1 produces the plots in Fig. 6 (C to E) of the main text, which show unit allocations in high-performing networks after completion of the algorithm, across different values of *s*.
