# Supplementary material for: Dual computational systems in the development and evolution of mammalian brains
Source: Sci Adv. 2026 Apr 22;12(17):eaec6112. doi: 10.1126/sciadv.aec6112 (PMC13101870; doi:10.1126/sciadv.aec6112)
Supplement: Supplementary file 1 — Supplementary Text Figs. S1 to S10 Table S1 [file sciadv.aec6112_sm.pdf]

Science Advances
AAAS

Supplementary Materials for

Dual computational systems in the development and evolution of mammalian brains

Nabil Imam *et al.*

Corresponding author: Nabil Imam, nimam6@gatech.edu; Barbara L. Finlay, blf2@cornell.edu

Sci. Adv. **12**, eaec6112 (2026)
DOI: 10.1126/sciadv.aec6112

This PDF file includes:

Supplementary Text
Figs. S1 to S10
Table S1

Covariation between limbic system and neocortex

Fig. S1a plots the combined size of all limbic system components (olfactory system, amygdala, hippocampus, schizocortex, and septum) and the neocortex against the size of core brain components (medulla, mesencephalon, diencephalon, striatum) across 182 mammalian species in 10 taxonomic groups. The residuals of each set of scatter points around their corresponding regression lines are plotted against one another in Fig. S1b, showing a robust negative covariation between the two structures.

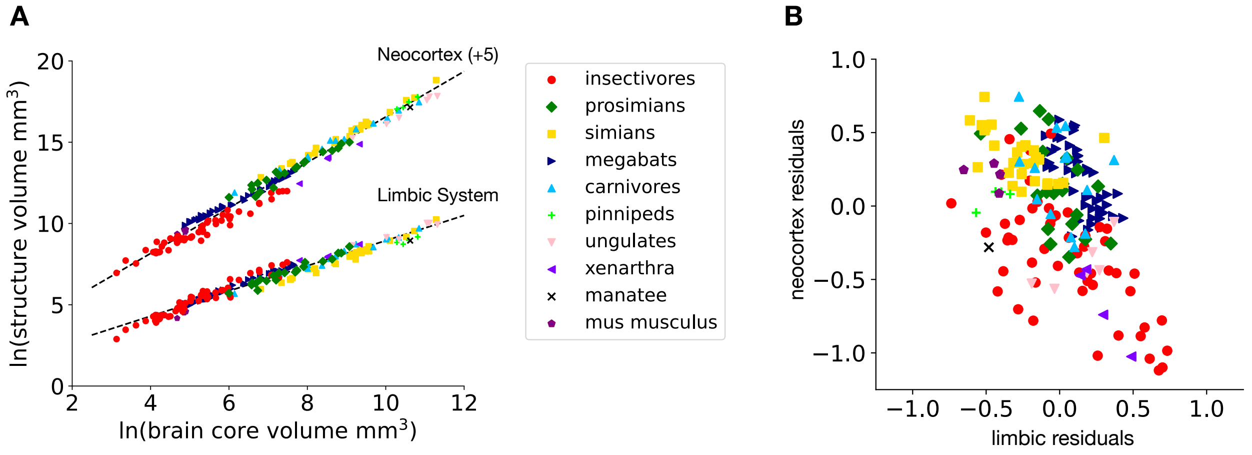

Figure S1: (a) Volumes of the neocortex and the limbic system plotted against the volume of core brain components across 182 species. An arbitrary constant of +5 is added to the neocortex data points to visually separate the plots. Both structures are strongly correlated with brain core ($r = 0.99$, $p < 0.001$ for neocortex; $r = 0.98$, $p < 0.001$ for limbic system). The allometric slope for the neocortex is $\boldsymbol{\beta} = 1.40$ (95% CI: 1.37-1.43), and for the limbic system is $\boldsymbol{\beta} = 0.77$ (95% CI: 0.75-0.80). (b) Residuals of the neocortex and limbic system data points in subplot *a* inversely covary with each other ($r = -0.50$, $p < 0.001$).

Allometric slopes and intercepts

The allometric slope and intercept of each structure examined in the main text is listed in Supplementary Table 1. All limbic system components have slopes less than 1 (hypoallomtric scaling), whereas the neocortex has a slope greater than 1 (hyperallometric scaling).

**Brain Structure**	**Slope (β)**	**Intercept (α)**
Neocortex	1.40 (1.37–1.43)	-2.44 (-2.66, -2.22)
Schizocortex	0.90 (0.87–0.92)	-1.58 (-1.76, -1.41)
Septum	0.77 (0.75–0.80)	-1.75 (-1.92, -1.57)
Hippocampus	0.83 (0.81–0.85)	-0.33 (-0.47, -0.18)
Amygdala	0.86 (0.84–0.88)	-1.62 (-1.49, -1.76)
Olfactory System	0.66 (0.63–0.69)	1.29 (1.07, 1.50)

Table 1: Allometric slopes and intercepts. 95% confidence intervals are indicated in brackets.

Imposing local connectivity

Fig. S2 shows the effects of imposing local connectivity in the olfactory and hippocampal networks prior to learning. The maps formed after learning remain disordered and a sharp drop in task accuracy is observed.

Local connectivity in the olfactory network is imposed by first assigning a one-dimensional physical position to each unit in every layer (position assignments were independent across layers). Each unit in the olfactory receptor neuron (ORN) layer is then connected to five neighboring units in the mitral cell (MC) layer (10% of units in the layer), with neighboring ORNs connecting to neighboring MCs. Similarly, each unit in the piriform cortex layer is connected to five neighboring units in the MC layer, with neighboring piriform neurons connecting to neighboring MCs.

Local connectivity in the hippocampal network of the Tolman-Eichenbaum Machine (TEM) is imposed by first assigning one-dimensional physical positions to each unit in the hippocampus and each unit in its sensory input layer (position assignments were independent across these two layers). Each unit in the input layer is then connected to neighboring units in the hippocampus (specifically to 10% of the units in each frequency module of the hippocampus). Neighboring units in the sensory layer connect to neighboring units (in each frequency module) of the hippocampus.

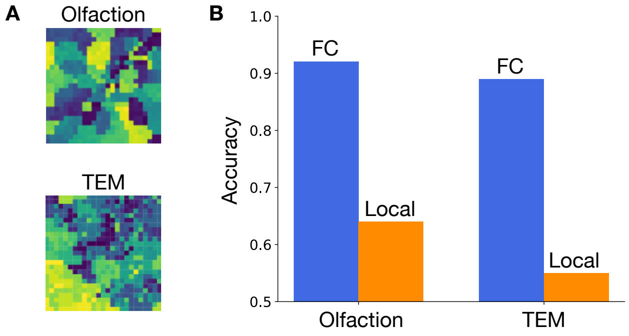

Figure S2: (a) Olfactory and hippocampal (TEM) maps generated from the representations of locally-connected networks. (b) Task accuracy achieved with fully-connected (FC) and locally-connected networks. For both olfaction and hippocampus, the fully-connected and locally-connected networks have the same number of trainable parameters.

Maps prior to model training

Fig. S3 shows the maps generated by the network models before they are trained on their respective datasets. Ordered maps can be seen for vision and somatosensation due to pre-configured locality in the network models. This is akin to the establishment of ordered spatiotopic maps and localized connections in primary visual and somatosensory cortices before sensory experience (17, 48). Audition lacks smooth maps despite pre-configured locality in the network model. Training the model generates smooth maps, as shown in Fig. 4a of the main text. For olfaction and hippocampus (TEM), maps generated by the untrained models are disordered and identical to the maps generated by trained models (Figs. 4a and 5d of the main text).

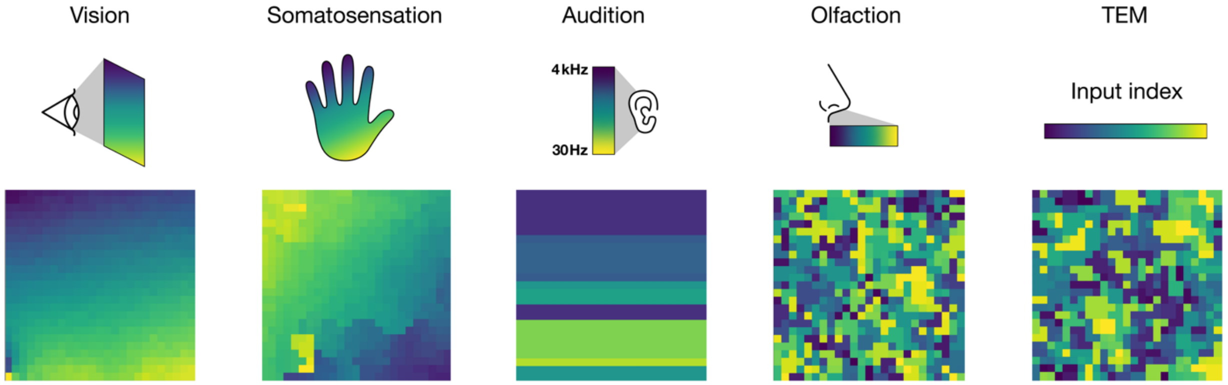

Figure S3: Maps generated by untrained models. The sensory surfaces are shown in the top row. Maps of those surfaces on model cortical sheets are shown in the bottom row. The maps are generated using the self-organizing map algorithm described in the main text.

Optimizing unstructured networks

To assess the patterns of information convergence that arise in artificial neural networks lacking pre-specified network structure, three-layer fully connected networks are trained using the same datasets used in the evolutionary algorithm (see Methods section of the main text). Each network is trained with the "***β***-LASSO" objective described in (53), which appends a weight thresholding step to stochastic gradient descent. This approach encourages network weights to trend aggressively towards zero during training, creating networks that are highly sparse, and which approximately match the performance of identical networks trained with stochastic gradient descent, while using only a fraction of the active (non-zero) parameters. The sparse connectivity resulting from this procedure is akin to resource-constrained connectivity in biological networks.

When evaluating these networks during training, we measure the level of sparsity in the first layer and tune the thresholding hyperparameter in **β**-LASSO to achieve a comparable number of active weights for each network. At the highest levels of sparsity (where 95-99% of weights are zero), the networks develop modality-specific patterns of connectivity with varying degrees of locality, reflecting the biases inherent in their respective datasets. Fig. S4 shows the distribution of receptive field sizes in each modality after tuning for this objective. Networks trained on data with a high degree of local correlation (vision, somatosensation and audition) generally have localized connectivity consisting of small clusters of non-zero weights, while those trained on data without locally correlated structure learn distributed patterns of connectivity across the full span of their inputs (olfaction and hippocampus). The receptive-field distributions in Fig. S4 recapitulate those observed in the main analyses (Figs. 4 and 5), demonstrating that the same localized versus distributed dichotomy emerges in initially unstructured networks.

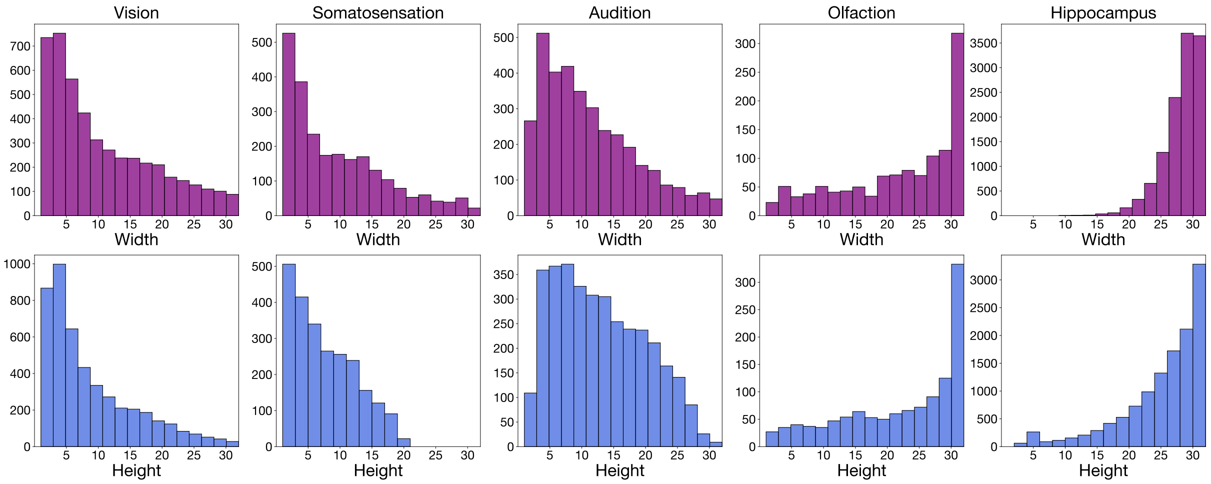

Figure S4: Distribution of receptive field sizes (height and weight shown separately) of network units after training a fully-connected network for each of the five modalities. The receptive field size of a unit is defined as the minimal bounding box surrounding all non-zero values of the unit's incoming weights arranged in two dimensions. Note the difference between the shape of the distributions in the spatiotopic modalities and olfaction/hippocampus.

Hyperparameter sweep of the self-organizing map algorithm

The configurable parameters in the self-organizing map algorithm (Algorithm 1 in main text) are the initial learning rate and the map size. All other parameters of the algorithm are computed as a function of these two. The results presented in the main text use an initial learning rate of 0.1 and a map size of 25 x 25. The results are consistent across variations of these settings. A parameter sweep is presented below.

Fig. S5 shows map order and receptive field locality indices for the five modalities across different values of the initial learning rate. The same patterns as in Figs. 5f-g of the main text are seen across these settings.

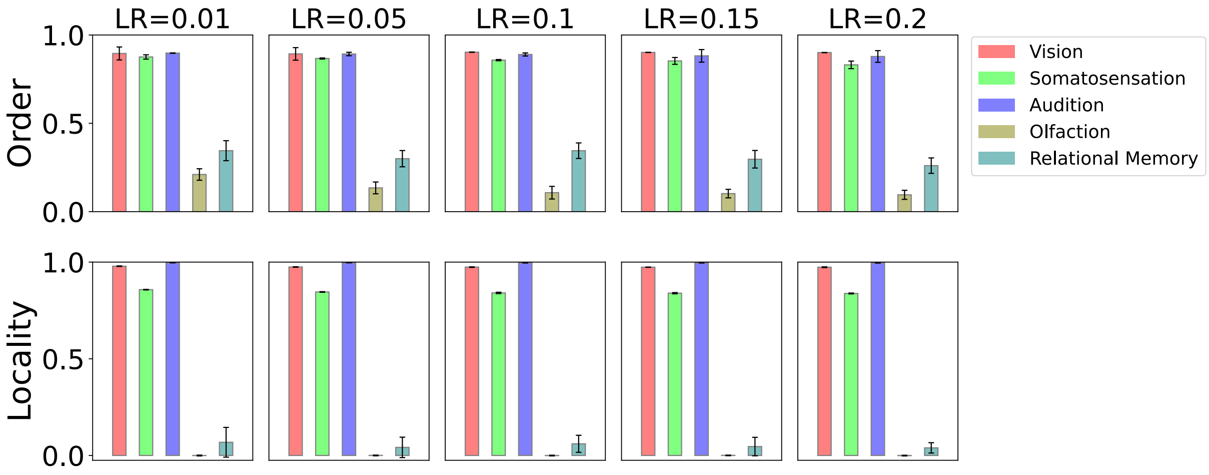

Figure S5: Spatiotopic order of the maps and the locality of their receptive fields in the five modalities across different values of the learning rate (LR). Plotted are the means and standard deviations across ten simulations with different random seeds.

Fig. S6 shows that different map sizes produce the same trends.

The same trends are also observed for different patch sizes (used to probe the organization of the map) as well as for PCA map initialization, where the initial weights of the map units are set to the first principal components of the map input instead of random values.

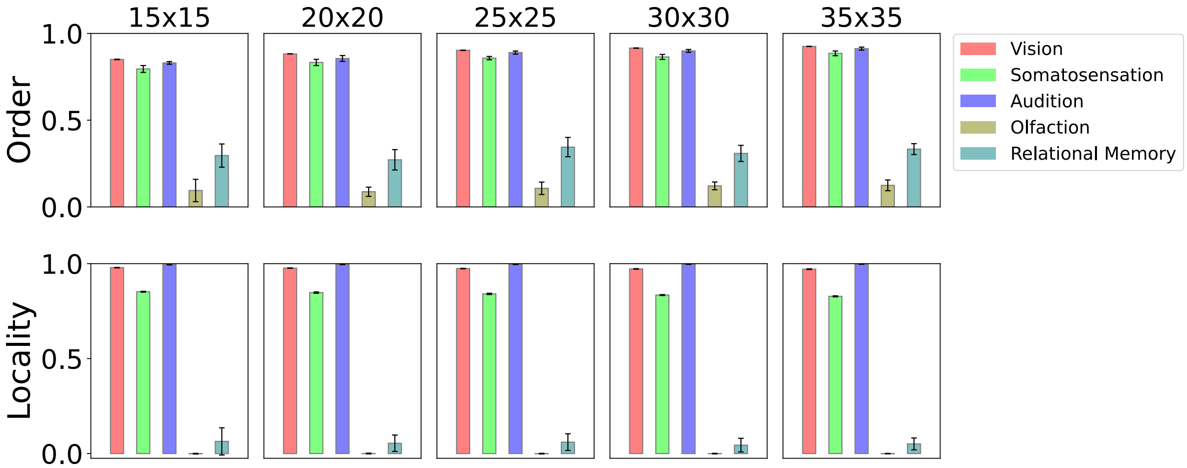

Figure S6: Spatiotopic order of the maps and the locality of their receptive fields in the five modalities across different map sizes. Plotted are the means and standard deviations across ten simulations with different random seeds.

Schematic of the evolutionary algorithm

Fig. S7 shows a schematic illustration of variation and selection in the evolutionary algorithm. A boundary *B* separates two distinct domains of network connectivity, one spatiotopic and the other distributed. Each domain consists of modality-specific networks: networks for vision, somatosensation, and audition in the spatiotopic domain, and networks for olfaction and relational memory (hippocampus) in the distributed domain. In the illustration of Fig. S7, the boundary location in the highest performing network (top row) of a generation is randomly shifted to create new networks. Among these new networks, the highest performing ones are selected for the next generation. Performance is measured by a combination of visual and olfactory performance determined by the parameter *s*, as described in the main text. In Fig. S7, olfactory performance is selected for (i.e., *s* is set to a low value). Consequently, the distributed domain expands at the expense of the spatiotopic domain. Note that other physical arrangements of the domains would lead to similar effects. For example, the networks can be placed from left to right in the following order: olfaction, spatiotopic modalities, hippocampus. The developmental variation in this arrangement would be an overall expansion (or contraction) of the spatiotopic domain, which would lead to a concerted contraction (or expansion) of the other two networks.

Hyperparameter sweep of the evolutionary algorithm

The configurable parameters in the evolutionary algorithm are (1) the fraction of the best-performing networks in a given generation that persist to the next generation, and (2) the extent of the variation of the boundary location around that of the highest-performing network. In the following, these two parameters are referred to as *k* and $\boldsymbol{\sigma}$ respectively. In Fig. 6 of the main text, values of k=0.5 and $\boldsymbol{\sigma}$=0.1 are used; $\boldsymbol{\sigma}$=0.1 means that new networks are formed by sampling uniformly within ±0.1 of the fittest network's boundary location, which lies in the range 0-1, 0 corresponding to one end of the network and 1 corresponding to the other end. The results are consistent across variations of these parameters. A parameter sweep is presented below.

Fig. S8 sweeps through different values of *k*. The pattern of covariation between network components stays the same across settings. Fig. S9 sweeps through different values of $\boldsymbol{\sigma}$, again showing the same trends.

When generating new networks, the total number of parameters allocated to the olfactory network is allowed to drift randomly by up to a percentage $\boldsymbol{\delta}$, resulting in an additional degree of variability among the new networks. In Fig. 6 of the main text, a value of $\boldsymbol{\delta}=10\%$ is used. The same overall trend is observed for different settings of $\boldsymbol{\delta}$, as shown in Fig. S10.

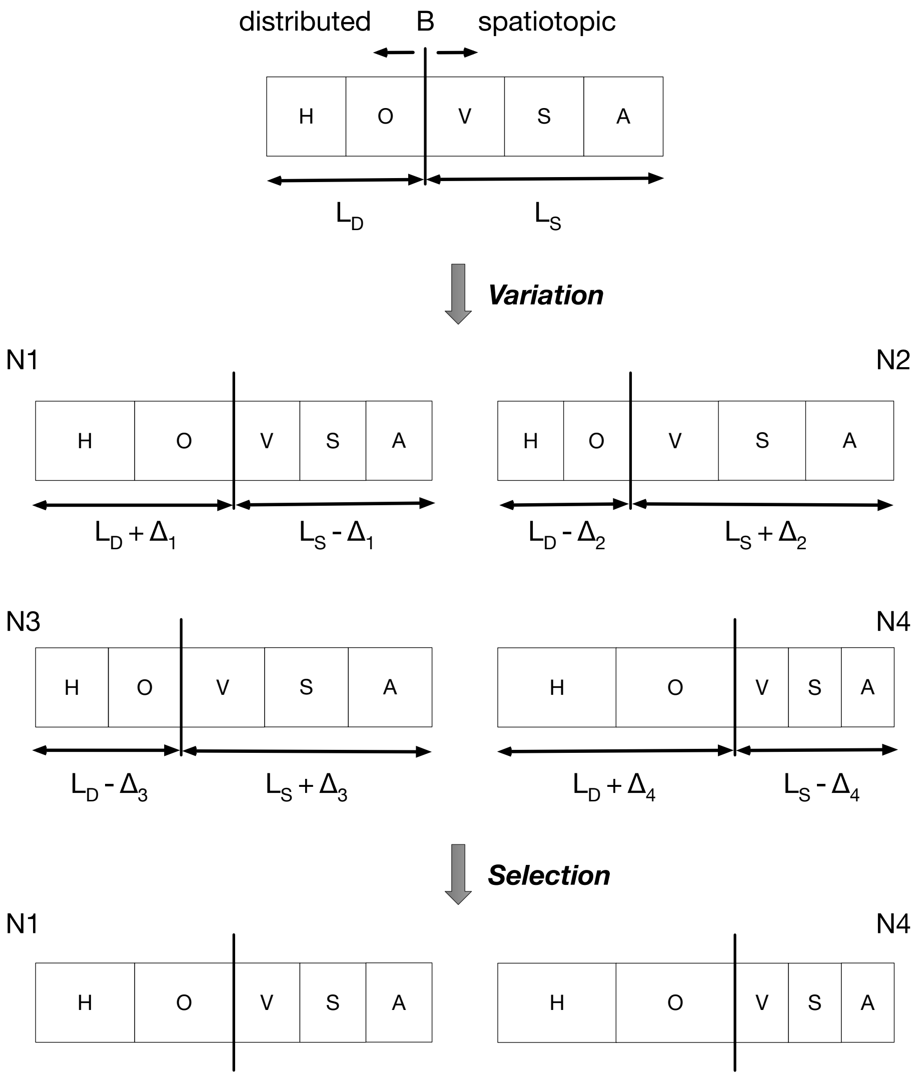

Figure S7: Illustration of variation and selection in the evolutionary algorithm. The top row shows the highest performing network of one generation. Variation of the boundary location in this network produces four new networks N1-N4. Among them, the networks with the highest olfactory performance are selected for the next generation. *H*: hippocampus, *O*: olfactory, *V*: vision, *S*: somatosensation, *A*: audition, *B*: boundary separating spatiotopic and distributed domains of connectivity, L_D: size of distributed domain, L_S size of spatiotopic domain. The **Δ** symbols here are positive constants denoting the variation of *B*.

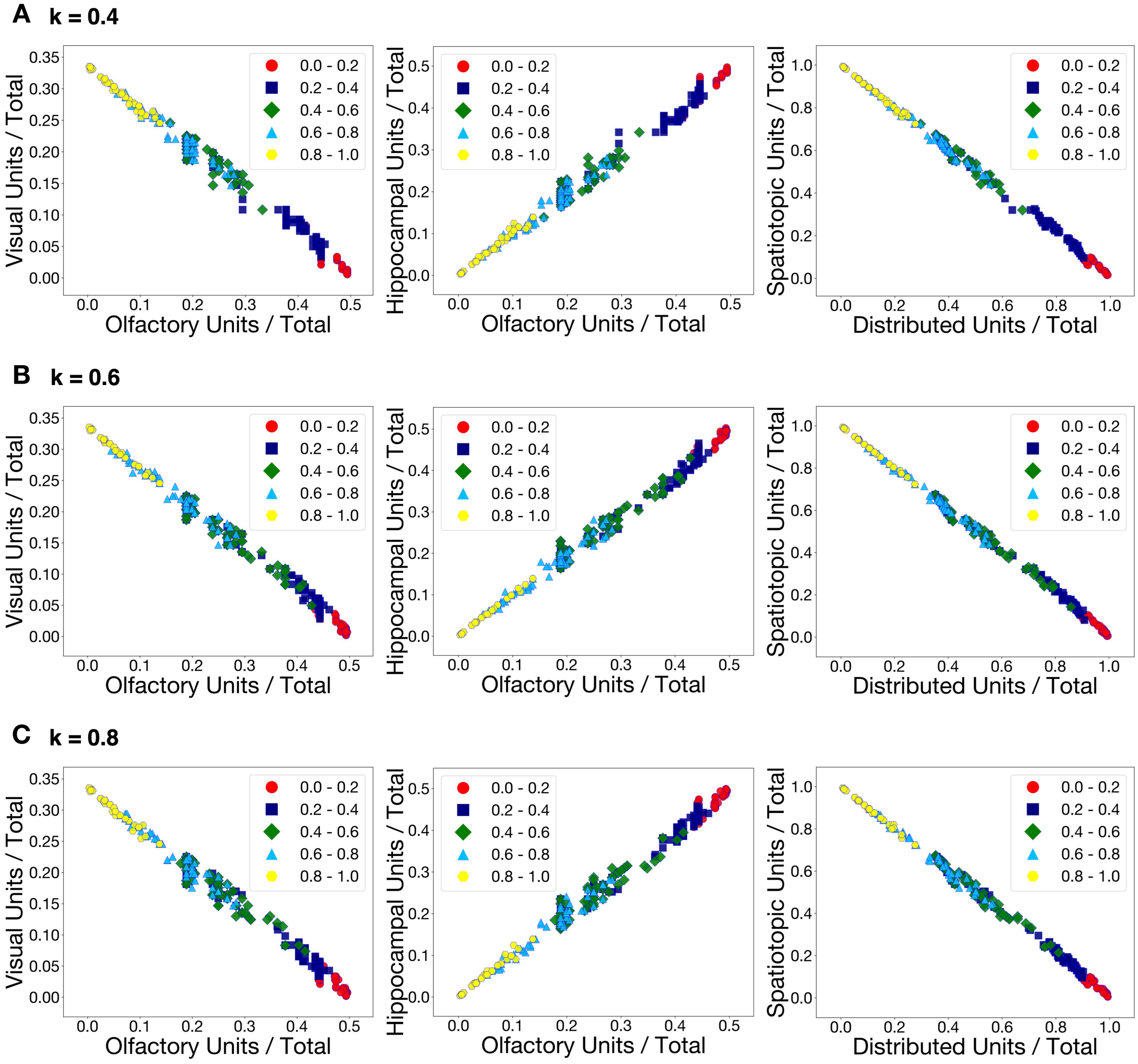

Figure S8: Covariation patterns resulting from the evolutionary algorithm for different values of the parameter k, which determines the fraction of best performing networks in a given generation that persist to the next generation.

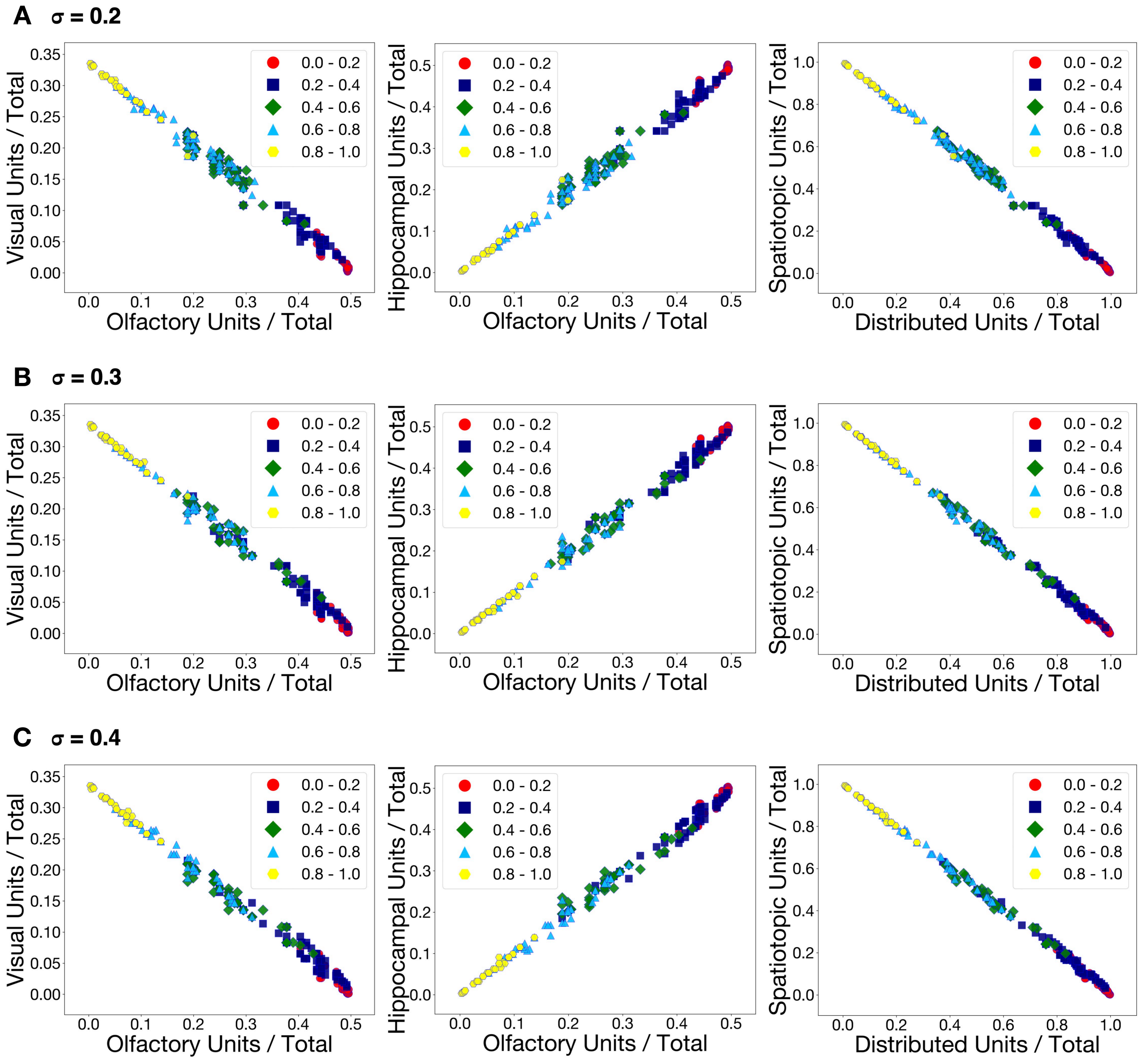

Figure S9: Covariation patterns resulting from the evolutionary algorithm for different values of the parameter $\boldsymbol{\sigma}$, which determines the extent of the variation of the boundary location around that of the highest-performing network.

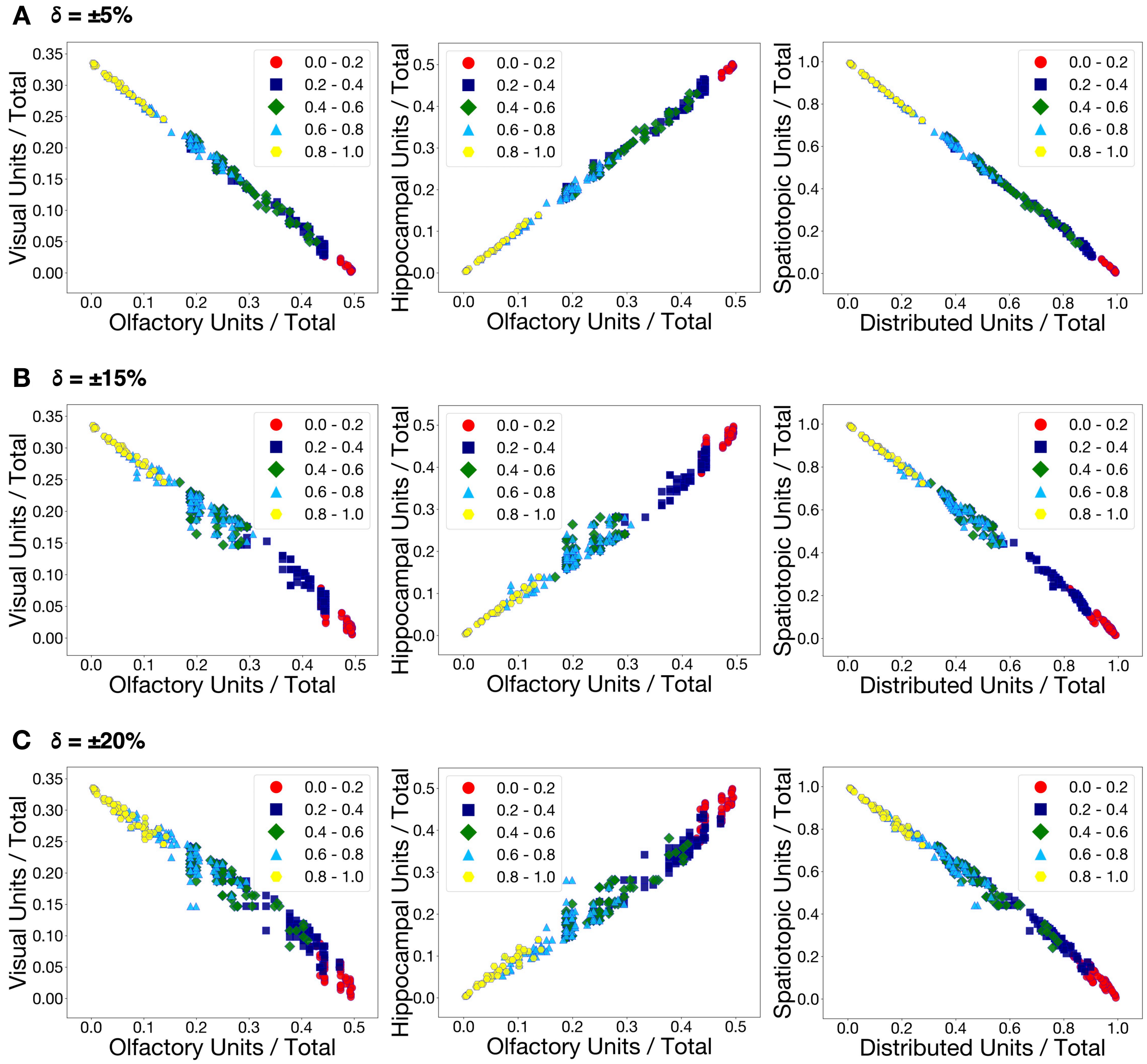

Figure S10: Covariation patterns resulting from the evolutionary algorithm for different values of the drift parameter **δ**.
